# Sand fly identification and screening for *Leishmania* spp. in six provinces of Thailand

**DOI:** 10.1186/s13071-021-04856-6

**Published:** 2021-07-03

**Authors:** Orawan Phuphisut, Chanyapat Nitatsukprasert, Nattaphol Pathawong, Boonsong Jaichapor, Arissara Pongsiri, Poom Adisakwattana, Alongkot Ponlawat

**Affiliations:** 1 Department of Helminthology, Faculty of Tropical Medicine, Mahidol University, Bangkok, 10400 Thailand; 2 Vector Biology and Control Section, Department of Entomology, Armed Forces Research Institute of Medical Sciences (AFRIMS), Bangkok, 10400 Thailand

**Keywords:** Sand fly, Phlebotomine, DNA barcoding, COI, *Leishmania* detection

## Abstract

**Background:**

Phlebotomine sand flies are vectors of *Leishmania* spp. At least 27 species of sand flies have been recorded in Thailand. Although human leishmaniasis cases in Thailand are mainly imported, autochthonous leishmaniasis has been increasingly reported in several regions of the country since 1999. Few studies have detected *Leishmania* infection in wild-caught sand flies, although these studies were carried out only in those areas reporting human leishmaniasis cases. The aim of this study was therefore to identity sand fly species and to investigate *Leishmania* infection across six provinces of Thailand.

**Methods:**

Species of wild-caught sand flies were initially identified based on morphological characters. However, problems identifying cryptic species complexes necessitated molecular identification using DNA barcoding in parallel with identification based on morphological characters. The wild-caught sand flies were pooled and the DNA isolated prior to the detection of *Leishmania* infection by a TaqMan real-time PCR assay.

**Results:**

A total of 4498 sand flies (1158 males and 3340 females) were caught by trapping in six provinces in four regions of Thailand. The sand flies were morphologically classified into eight species belonging to three genera (*Sergentomyia*, *Phlebotomus* and *Idiophlebotomus*). *Sergentomyia iyengari* was found at all collection sites and was the dominant species at most of these, followed in frequency by *Sergentomyia barraudi* and *Phlebotomus stantoni*, respectively. DNA barcodes generated from 68 sand flies allowed sorting into 14 distinct species with 25 operational taxonomic units, indicating a higher diversity (by 75%) than that based on morphological identification. Twelve barcoding sequences could not be assigned to any species for which cytochrome *c* oxidase subunit I sequences are available. All tested sand flies were negative for *Leishmania* DNA.

**Conclusions:**

Our results confirm the presence of several sand fly species in different provinces of Thailand, highlighting the importance of using DNA barcoding as a tool to study sand fly species diversity. While all female sand flies tested in this study were negative for *Leishmania*, the circulation of *Leishmania* spp. in the investigated areas cannot be ruled out.

**Graphical abstract:**

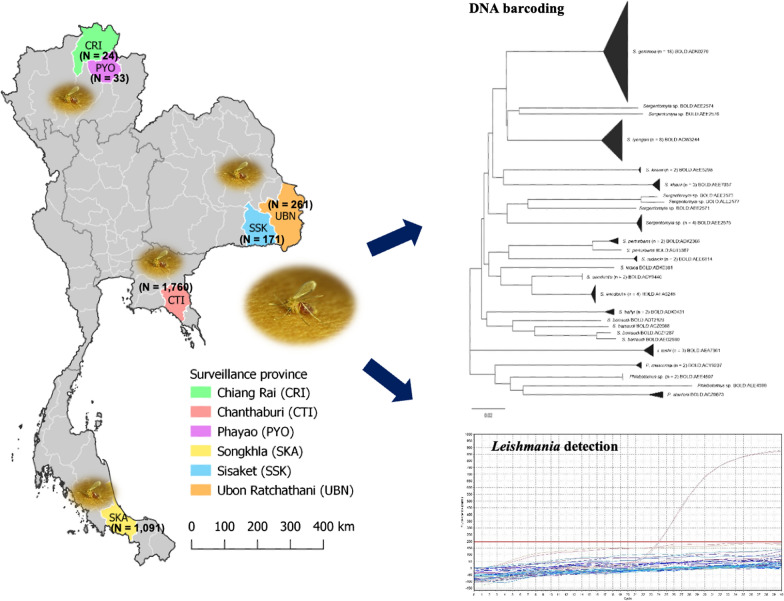

**Supplementary Information:**

The online version contains supplementary material available at 10.1186/s13071-021-04856-6.

## Background

Leishmaniasis is a neglected tropical disease caused by flagellate protists of the genus *Leishmania*, which are transmitted by the bite of infected female phlebotomine sand flies (Diptera: Psychodidae: Phlebotominae) [1]. This vector-borne disease is endemic in Central and North America, the Indian subcontinent, the Mediterranean basin, the Middle East and Central Asia. More than a billion people distributed across more than 98 countries worldwide are at risk of infection [2]. Leishmaniasis is responsible for 20,000−30,000 deaths annually, and there are an estimated 1.3 million new cases each year, with 30,000 new cases of visceral leishmaniasis (VL) and more than a million new cases of cutaneous leishmaniasis (CL) [3]. CL, the most common form of leishmaniasis, causes skin sores, while VL or kala-azar, the most severe form, affects the internal organs, including the spleen, liver and bone marrow [4, 5]. Currently, no vaccine is available for leishmaniasis [6–8]. The prevention and control of leishmaniasis require a combination of intervention strategies [9–11]. Vector control is an important strategy for disease prevention, as it reduces and interrupts the transmission of this disease by reducing the density of sand fly populations [12].

More than 20 *Leishmania* parasite species are known to be infective to humans [13]. *Leishmania martiniquensis* and *L. orientalis* (formerly *L. siamensis*) [14] are causative agents of CL and VL in Thailand. Although Thailand is not considered to be an endemic country for leishmaniasis, since 1999 there has been an increase in the number of reported cases of autochthonous leishmaniasis in several regions of the country [15]. A recent review of sand fly distribution in Thailand indicated that at least 27 species of the genera *Sergentomyia*, *Phlebotomus*, *Idiophlebotomus* and *Chinius* have been identified [16, 17]. *Sergentomyia gemmea* is the predominant species in Thailand and is considered to be a potential vector of *L. orientalis* [18]. However, there are some reports of the detection of the DNA of *Leishmania* parasites in sand flies, including *L. martiniquensis* DNA in *S. gemmea*, *S. barraudi* and *S. khawi* [15, 18, 19] as well as in black rats (*Rattus rattus*) [15]. Moreover, *L. orientalis* DNA was detected in *S. iyengari* [20]. These studies were conducted in areas of southern Thailand where human leishmaniasis cases have been recorded [18, 21]. However, little is known regarding species richness, distribution and vector role of sand flies in other regions of Thailand.

Given this background, the aim of our study was to investigate the distribution and identity of sand fly species in six provinces of four different regions of Thailand. We also investigated the presence of *Leishmania* DNA in female sand flies.

## Methods

### Study sites

**Fig. 1:**
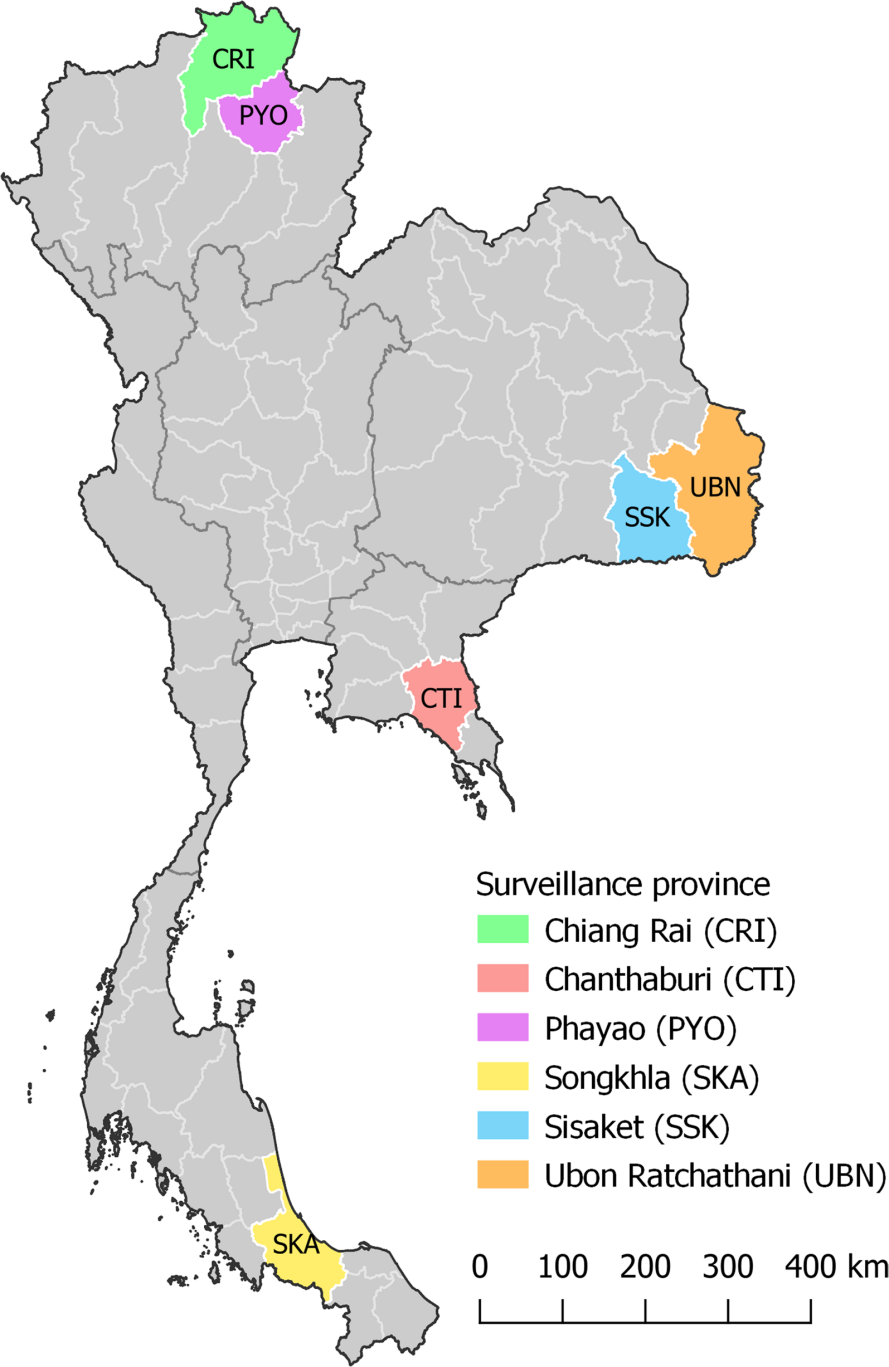
Map showing the locations of the six provinces in Thailand where the surveillance of sand flies was conducted: Chiang Rai (*CRI*), Phayao (*PYO*), Chantaburi (*CTI*), Ubon Ratchathani (*UBN*), Sisaket (*SSK*), and Songkhla (*SKA*)

The distribution of sand fly species and their potential as vectors of leishmaniasis were assessed in six provinces distributed in four regions of Thailand, as follows: (i) northern region: Chiang Rai (CRI) and Phayao (PYO) provinces; (ii) northeastern region: Sisaket (SSK) and Ubon Ratchathani (UBN) provinces; (iii) eastern region: Chantaburi (CTI) province; and (iv) southern region: Songkhla (SKA) province (Fig. 1). The study sites were selected based on a previous report of human leishmaniasis cases in three of the provinces (CTI, CRI and SKA) [15]. The other study areas, where no cases of leishmaniasis have been reported to date (including PYO, SSK, and UBN), were selected based on tourist attractions, including national parks and limestone caves. The collecting of sand flies was conducted in 12 villages located in eight districts, with collection sites including the outdoor areas of surrounding houses, enclosures, animal pens, barns and rubber tree and banana tree plantations. Similarly, surveillance was conducted inside three tourist caves, namely Tham Pajom, Tham Ho and Tham Khao Roopchang, located in CRI, PYO and SKA provinces, respectively. All caves were limestone caves and surrounded by a mixed deciduous forest, and they are inhabited by many bats. Average temperature and location coordinates of the study sites were recorded (Additional file 1: Table S1). Rainfall data were provided by the Thai Meteorological Department.

### Sand fly collection and morphological identification

From November 2016 to January 2018, CDC light traps augmented with CO_2_ were placed at the chosen collecting areas, at a height of approximately 50 cm above the ground, and operated continuously for 14 h (18:00 h to 08:00 h). Specimens were kept in dry ice and transported to the laboratory of the Armed Forces Research Institute of Medical Sciences (AFRIMS), Bangkok, Thailand.

Female sand flies were sorted and used for species identification based on their morphology, according to Lewis [22]. Each female was separated into three parts, i.e. the head, body (thorax and abdomen) and posterior segments of the abdomen (containing the spermatheca). The head and the posterior segments of the abdomen were mounted on permanent slides using a standard protocol for sand fly speciation [23]. The specimens were examined under a compound microscope (Olympus, Tokyo, Japan). The remaining body parts (thorax and abdomen) were kept at − 80 °C for analysis of DNA barcoding and *Leishmania* detection.

### Molecular identification of sand flies using DNA barcoding

In addition to morphological analysis, representative specimens of each sand fly species (99 specimens) identified on the basis of morphological characters were subjected to molecular identification using DNA barcoding. DNA was extracted from the stored body parts using DNAzol^®^ reagent (Molecular Research Center, Cincinnati, OH, USA). In detail, specimens were homogenized in 500 µl of DNAzol^®^ reagent, and genomic DNA was isolated from the homogenate according to the manufacturer’s instructions, following which it was resuspended in 30 µl of nuclease-free water. The DNA barcode region of the cytochrome *c* oxidase I (COI) region [24, 25] was amplified using standard protocols and primers for insect DNA barcoding, namely the forward (Fw) primer LCO1490 (5′-GGTCAACAAATCATAAAGATATTGG-3′) and the Rv primer HCO2198 (5′-TAAACTTCAGGGTGACCAAAAATCA-3′) [26]. The PCR reaction consisted of 1 μl of total DNA mixed in 12.5 μl of 2 × *TopTaq* Master Mix (Qiagen, Hilden, Germany) and 10 μM Fw and Rv primers, to a final volume of 25 μl. The thermocycling conditions consisted of: 94 °C, 5 min for 1 cycle; then 94 °C/40 s, 52 °C/4 s, 72 °C/1 min for 40 cycles; and a final extension at 72 °C for 5 min. The expected amplicons of the PCR products (648 bp) were analyzed in 1.5% agarose gel mixed with 1 × SYBR Safe DNA gel stain (Invitrogen, Thermo Fisher Scientific, Waltham, MA, USA). The gels were visualized and photographed using the Gel Doc XR System (Bio-Rad, Hercules, CA, USA). The PCR products were purified using the QIAquick Gel Extraction Kit (Qiagen) prior to DNA sequencing (Celemics, Inc., Seoul, Korea).

### DNA barcode analysis

The DNA barcodes were analyzed according to the method described by Wilson et al. [26] and deposited in the Barcode of Life Data System (BOLD) [27] in the project “OPHU”. A neighbor-joining (NJ) tree was constructed and genetic distances calculated using MEGA X [28] and the Kimura two-parameter (K2P) model with default settings [29]. The DNA barcodes were assigned to operational taxonomic units (OTUs) using the Refined Single Linkage (RESL) algorithm based on their Barcode Index Number (BIN) [27] assignment in BOLD. A barcode gap analysis was performed and intra-OTU distances (K2P) were calculated using BOLD and MEGA X.

### Detection of *Leishmania* DNA among sand flies by theTaqMan real-time PCR assay

Specimens belonging to the genera *Phlebotomus*, *Sergentomyia* and *Idiophlebotomus* were sorted and pooled by species based on the morphological identification and capture locations. In total, 33 pools of *Phlebotomus* (131 females), 200 of *Sergentomyia* (3203 females) and one pool of *Idiophlebotomus* (6 females) were investigated. Each pool contained between 10 and 20 female sand flies. DNA was isolated from each pool as mentioned above and subsequently used for the detection of *Leishmania* infection by TaqMan real-time PCR. Primers and probes were selected from DNA polymerase 2 to detect all *Leishmania* species, as described in a previous study [30]. The primers (AF009136) consisted of a Fw primer (5′-AGGAGGATGGCAAGCGGAAG-3′) and a Rv primer (5′-GCGACGGGTACAGGGAGTTG-3′). The TaqMan probe (5′-FAM-TGGGGTCGAGCACCATGCCGCC-TAMRA-3′) was labeled with 5′FAM and 3′TAMRA as the reporter and quencher, respectively. All reactions were conducted on a MasterCycler RealPlex4 instrument (Eppendorf, Hamburg, Germany) in 10-µl reaction mixtures containing 5 µl of *iTaq* Universal Probes supermix (Bio-Rad), 200 nM each of the Fw and Rv primers, 50 nM TaqMan probe and 1 µl of DNA template (approx. 1 µg). The thermocycling conditions consisted of a hold at 95 °C, 2 min; followed by 95 °C/5 s and 60 °C/1 min for 40 cycles. A cut-off of 35 cycles was used to define positive samples with a fluorescence signal above the background noise. The detection of each pooled DNA was performed in duplicate. The detection limit of the assay was established by spiking tenfold serial dilutions of *Leishmania* DNA into the genomic DNA of sand flies, to a final concentration ranging from 10 ng/µl to 1 fg/µl. The threshold cycle values were determined by the optimum standard curve produced by dilutions of the *Leishmania* DNA.

According to the negative results obtained by TaqMan real-time PCR, the detection of *Leishmania* DNA in all sand fly specimens was also performed using a conventional PCR method targeting the ITS1 region as described in [31]. Briefly, the reactions were performed using primers LeF (5′-TCCGCCCGAAAGTTCACCGATA-3′) and LeR (5′-CCAAGTCATCCATCGCGACACG-3′). A PCR reaction volume consisted of 1 μl of total DNA mixed with 12.5 μl of 2 × *TopTaq* Master Mix and 10 μM each of the Fw and Rv primers, to a final volume of 25 μl. The thermocycling conditions consisted of 95 °C, 5 min; followed by 95 °C/1 min, 50 °C/1 min, 72 °C/1 min for 40 cycles; and a final extension at 72 °C for 7 min. The PCR products were analyzed by electrophoresis and visualized using the Gel Doc XR System (Bio-Rad).

## Results

### Sand fly collection

A total of 4498 sand flies (1158 males and 3340 females) were captured by trapping during 443 CDC light trap nights. The greatest number of captured sand flies were collected from Chantaburi province (2251 specimens), followed by Songkhla province (1593 specimens), Ubon Ratchathani province (336 specimens), Sisaket province (226 specimens), Phayao province (49 specimens) and Chiang Rai province (43 specimens), as shown in Table 1. In CTI province most of the sand flies (491 males and 1760 females) were trapped at a location surrounded by rubber tree plantations. With respect to the three cave locations, the greatest number of sand flies were caught at Tham Ho (49 specimens) in Phayao Province, followed by Tham Khao Roopchang (37 specimens) in Songkhla province and Tham Pajom (43 specimens) in Chiang Rai province.

**Table 1:** Number of sand flies collected by CDC light traps with CO_2_

Locations	Number of males	Number of females	Total number	Total traps	Male catch rate/trap	Female catch rate/trap
Chantaburi (CTI)	491	1760	2251	136	3.6	12.9
Chiang Rai (CRI)	19	24	43	22	0.9	1.1
Phayao (PYO)	16	33	49	22	0.7	1.5
Sisaket (SSK)	55	171	226	100	0.6	1.7
Songkhla (SKA)	502	1091	1593	123	4.1	8.9
Ubon Ratchathani (UBN)	75	261	336	40	1.9	6.5
Total	1158	3340	4498	443	2.6	7.5

The head and abdomen of 3340 female sand flies were morphologically identified under a compound microscrope to establish the species. Based on morphological identification, the sand flies were classified into eight species belonging to three genera: *Sergentomyia*, *Phlebotomus* and *Idiophlebotomus* (Table 2), of which 24 specimens were classified only to the genus level due to unclear characters of the cibarium and the spermatheca on the slides; these 24 specimens were thus further identified using DNA barcoding. The most common species in this study was *Sergentomyis iyengari* (2970 specimens), which was found at all collection sites, followed by *S. barraudi* (129 specimens) and *Phlebotomus stantoni* (123 specimens).

**Table 2:** Sand fly species identified in six provinces of Thaliand in this study, based on morphological and DNA barcoding analyses of female specimens

Sand fly identity	Provinces						Total	Number of DNA barcodes	Maximum intra-OTU
	CTI	CRI	PYO	SSK	SKA	UBN			
*Idiophlebotomus teshi*	–	6	–	–	–	–	6	3	0.41
*Phlebotomus mascomai*	–	–	2	–	–	–	2	2	0.54
*Phlebotomus* spp.	–	–	–	–	3	–	3	0	N/A
*Phlebotomus* sp. (D00059, D00062)	–	–	–	2	–	–	2	2	0.00
*Phlebotomus* sp. (D00103)	–	–	–	1	–	–	1	1	0.00
*Phlebotomus stantoni*	1	5	26	17	65	9	123	2	1.44
*Sergentomyia anodontis*	–	–	1	–	–	–	1	0	N/A
*Sergentomyia anodontis *(D00014, D00015, D00017, D00018)	–	–	–	–	4	–	4	4	0.36
*Sergentomyia anodontis *(D00079, D00089)	–	2	–	–	–	–	2	2	0.00
*Sergentomyia bailyi*	2	–	–	–	–	–	2	2	1.09
*Sergentomyia barraudi*	5	–	–	–	–	–	5	0	N/A
*Sergentomyia barraudi* (D00016)	–	–	–	–	45	–	45	1	0.00
*Sergentomyia barraudi* (D00092)	–	–	2	–	–	–	2	1	0.00
*Sergentomyia barraudi *(D00047, D00065)	–	–	–	49	–	28	77	2	1.99
*Sergentomyia gemmea*	13	–	–	1	3	2	19	18	2.58
*Sergentomyia indica*	4	–	–	4	17	4	29	1	0.00
*Sergentomyia iyengari*	1,728	2	2	91	936	211	2,970	8	2.16
*Sergentomyia khawi *(D00060, D00061)	–	–	–	2	–	–	2	2	0.18
*Sergentomyia khawi *(D00074, D00086, D00088)	–	3	–	–	–	–	3	3	0.00
*Sergentomyia perturbans *(D00021, D00111)	1	–	–	–	1	–	2	2	0.64
*Sergentomyia perturbans* (D00027)	–	–	–	–	1	–	1	1	0.00
*Sergentomyia rudnicki*	–	2	–	–	–	–	2	2	0.18
*Sergentomyia* spp.	6	–	–	3	15	4	28	0	N/A
*Sergentomyia* sp. (D00009)	–	–	–	–	1	–	1	1	0.00
*Sergentomyia* sp. (D00042)	–	–	–	–	–	1	1	1	0.00
*Sergentomyia* sp. (D00050)	–	–	–	–	–	1	1	1	0.00
*Sergentomyia* sp. (D00055)	–	–	–	–	–	1	1	1	0.00
*Sergentomyia* sp. (D00058)	–	–	–	1	–	–	1	1	0.00
*Sergentomyia* sp. (D00069, D00070, D00075, D00078)	–	4	–	–	–	–	4	4	0.35
Total	1760	24	33	171	1091	261	3340	68	

Only 68 sequences underwent DNA barcode analysis

*N/A* Not applicable, *OTU* operational taxonomic unit

### DNA barcode analysis

A total of 99 specimens of the 3340 morphologically identifiable females possessed representative morphological characters. Of these 99 specimens, 75 females were morphologically classified to species level, and 24 were only morphologically identifiable to the genus level. Only 74 of of the 99 specimens identified to species level were successfully amplified using the DNA barcode primers. Six of these 74 sequences were excluded from the analysis because they were ambiguous; therefore, 68 sequences underwent further DNA barcode analyses (Table 2). The DNA barcodes were sorted according to the cluster analysis into 14 species with 25 OTUs, which represented a significant increase from the eight species identified based on morphology.

The 24 specimens that were morphologically identified only to the genus level were analyzed by DNA barcoding; of these, 12 could be identified to the species level with > 98% identity: *Phlebotomus mascomai* (*n* = 2), *Sergentomyia bailyi* (*n* = 2), *S. gemmea* (*n* = 3), *S. perturbans* (*n* = 3) and *S. rudnicki* (*n* = 2). The remaining 12 specimens were still only identifiable to the genus level: *Phlebotomus* sp. (*n* = 3) and *Sergentomyia* sp. (*n* = 9).

Interestingly, four sand fly species were sorted into multiple clusters by the DNA barcode analysis, including *S. anodontis* (2 OTUs), *S. barraudi* (3 OTUs), *S. khawi* (2 OTUs), and *S. perturbans* (2 OTUs), with a maximum intraspecific distance of 0.4, 2.0, 0.2 and 0.6%, respectively (Additional file 2: Table S2). According to the barcode gap analysis, six of 14 species had a maximum intraspecific distance of > 3%, including *Sergentomyia* sp., *Phlebotomus* sp., *S. khawi*, *S. barraudi*, *S. anodontis* and *S. perturbans* (Additional file 3: Table S3). The top three highest maximum intraspecific distances were identified for *Sergentomyia* sp. (21.0%), followed by *Phlebotomus* sp. (17.2%) and *S. khawi* (17.0%).

**Fig. 2:**
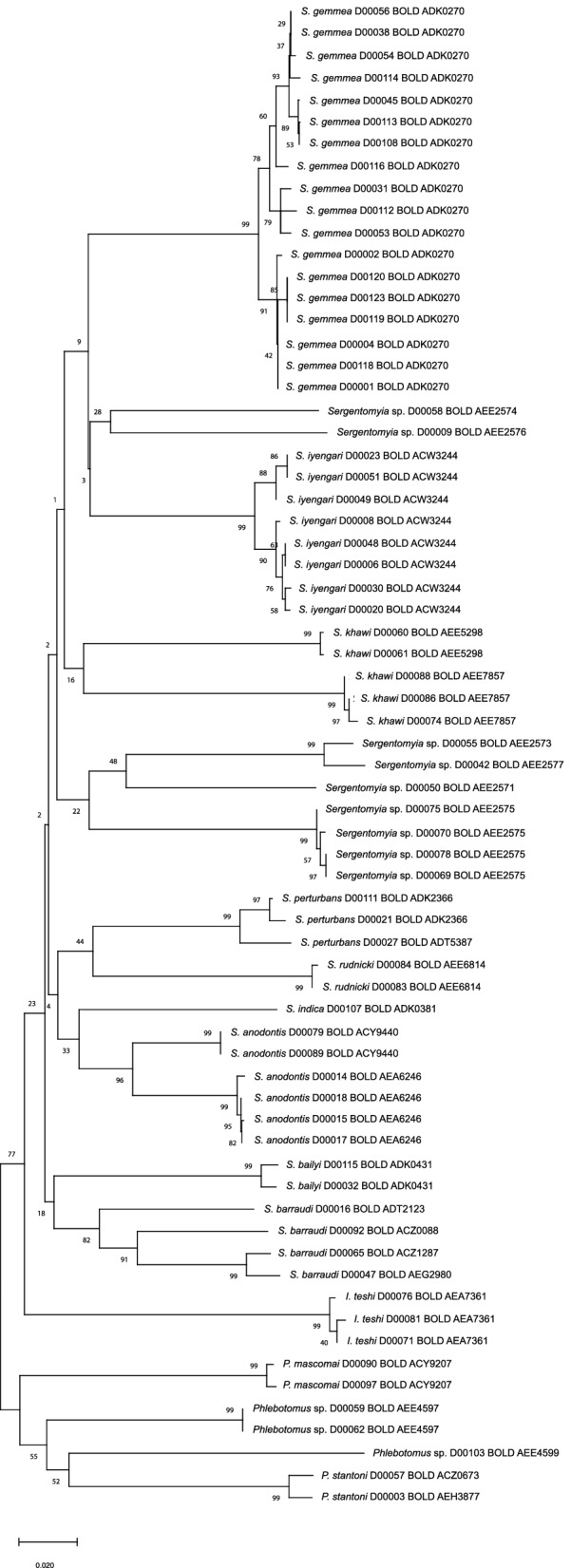
Neighbor-joining tree based on the cytochrome *c* oxidase I (COI) sequences using the Kimura 2-parameter (K2P) distance in MEGA X

The NJ tree of K2P distances (Fig. 2) demonstrates a clear separation into two clades of *Sergentomyia* and *Idiophlebotomus* (clade I) and *Phlebotomus* (clade II). *Idiophlebotomus* was included in clade I as a sister group of *Sergentomyia*, of which the nearest neighbor was *S. anodontis* with a genetic distance of 0.1627 (Additional file 4: Table S4). According to genetic distance, the variation within each species indicated that *S. khawi* and *S. rudnicki* had the highest and the lowest intraspecific distance among other species, with a genetic distance of 0.1771 and 0.0018, respectively. The nearest neighbor was found for *S. anodontis* (D00079 and D00089) and *S. indica* (D00107), with a genetic distance of 0.1184.

### *Leishmania* DNA detection in sand flies

The TaqMan real-time PCR assay performed in this study was capable of detecting *Leishmania* DNA at a level as low as 1 pg. However, no *Leishmania* DNA was detected in any of the sand fly samples. The amplification curve generated by MasterCycler RealPlex4 revealed a cumulative fluorescent signal of *Leishmania* DNA in the reaction (Additional file 5: Figure S1). Because of the negative results obtained by the TaqMan real-time PCR assay, the detection of *Leishmania* DNA in all sand fly specimens was also performed using a conventional PCR method, with negative results.

## Discussion

Based on the morphological identification of female specimens, *S. iyengari* was the predominant species in several of the provinces investigated in this study, especially Chantaburi, Songkhla and Ubon Ratchathani provinces. The results of previous studies suggested that the species richness of sand flies in Thailand varied greatly among different provinces or habitats [19, 32–36], possibly indicating that sand fly richness in Thailand might exhibit an area-specific distribution. These results indicated the need to investigate sand fly richness in several regions and habitats in order to obtain reliable information on sand fly species richness in Thailand.

In addition to area, seasonal variation may impact on sand fly density, as previously observed for *S. silvatica* and *P. argentipes* densities in Saraburi province, Thailand, which varied with the season [34]. Moreover, the density of cave-dwelling sand flies surveyed in Uthai Thani province, Thailand fluctuated during the year, with the highest peak in December (28.5%) and the lowest in May (2.3%) [35]. Because sand fly collections from all sites in this study were performed in the same season (November–January), further investigations of seasonal variation of sand fly densities at these sites are required.

The identification of sand flies is traditionally achieved based on their morphology, primarily through microscopic observation of head and genitalia characters [37], a process which requires taxonomic expertise [38]. Both phenotypic plasticity and cryptic species complexes pose further challenges for the identification of sand fly species based on morphology [39]. These obstacles have led to the increased application of DNA barcoding analysis for the identification of species of sand flies in several geographical areas [38–43], including Thailand [17]. In the present study, DNA barcoding analysis was performed to identify the species of the 24 female sand fly specimens that could be classified only to the genus level based on morphological characters; 12 of these 24 specimens were subsequently identified using DNA barcoding to the species level, including *P. mascomai*, *S. bailyi*, *S. gemmea*, *S. perturbans* and *S. rudnicki*; however, the remaining 12 specimens were still identified to the genus level only. These unclassified species may be newly recorded species; however, an analysis using additional DNA markers, such as the *Cytb* gene [16, 44], is warranted prior to reaching a definitive conclusion. The DNA barcoding showed that five (11.4%) of the 44 specimens identified to species level on the basis of morphological chararacters actually belonged to a different species. In particular, five of 13 specimens morphologically identified as *S. iyengari* were actually *S. gemmea*; the remaining eight specimens were indeed *S. iyengari*. As such, the possibility that some of the females identified as *S. iyengari* (see Table 2) were actually *S. gemmea* cannot be ruled out. The morphology of the cibarium is the key difference between *S. iyengari* and *S. gemmea*. The cibarium of *S. iyengari* females exhibits central teeth that are smaller than the other teeth, and the fore teeth are absent or range from one row of four teeth to two rows of up to 20 teeth; in contrast, the cibarium of female *S. gemmea* has ten hind teeth with broad bases narrowing abruptly to fine points, with one row of eight very large fore teeth or two rows of small teeth in front of them [22]. The misidentification of some specimens might be attributed to the quality of the sand fly slides, which led to unclear characters of the cibarium and the spermatheca. Moreover, a specific morphological key of sand flies in Thailand and the South East Asia region has never been developed. A sand fly key for this region is greatly needed to achieve a greater accuracy of morphological identification. Because of this lack of a sand fly key for Thailand and the surroundings region, molecular tools such as DNA barcoding analysis are tremendously helpful in morphological identification.

All specimens for sequencing were obtained from female sand flies. After phylogenetic clustering, four species of sand fly were divided into multiple clusters, including *S. anodontis* (two OTUs), *S. barraudi* (three OTUs), *S. khawi* (two OTUs) and *S. perturbans* (two OTUs), with a similarity among OTUs < 94%, < 92%, < 85%, and < 94%, respectively. These findings suggest an unexpectedly high genetic variation in DNA barcodes, possibly indicating the presence of cryptic species. For example, there have been reports of cryptic species of *S. barraudi* caught in a tourist cave, Uttaradit Province, northern Thailand, as revealed by DNA barcoding [20, 45].

The barcode analysis performed in this study split four DNA barcode sequences of *S. barraudi* into three OTUs assigned into four BINs, i.e. BOLD:ACZ0088, BOLD:ACZ1287, BOLD:ADT2123 and BOLD:AEG2980. Two OTUs (BOLD:ACZ0088 and BOLD:ACZ1287) that had been reported by Sukantamala et al. in 2017 [45] were also found in this study, i.e. *S. barraudi* D00092 from Phayao Province and *S. barraudi* D00065 from Sisaket Province. Our results support the contention that *S. barraudi* formed a species complex [21, 45]. Moreover, two members of one OTU were split into two BINs, i.e. BOLD:AEG2980 (*S. barraudi* D00047 from Ubon Ratchathani Province) and BOLD:ACZ1287 (*S. barraudi* D00065 from Sisaket Province). The additional OTU found in this study was *S. barraudi* D00016 from Songkhla Province, which was assigned into BOLD:ADT2123. However, this OTU was a singleton containing only a barcoding sequence of *S. barraudi* D00016; therefore, additional specimens need to be found to confirm the existence of this OTU. In the future, these species require further taxonomic investigation to support the current findings, specifically to determine whether they are different cryptic taxa or are indications of geographic structuring within a single species.

In Thailand, *S. gemmea*, *S. barraudi*, *S. khawi* and *S. iyengari* act as potential *Leishmania* vectors as they have been found to be positive for the presence of *L. martiniquensis* and *L. orientalis* in human leishmaniasis transmission areas [18–21]. However, the sand flies caught in this study were found not to be infected with *Leishmania*. These negative results suggest that more studies involving more traps per area need to be conducted with the aim to collect a large number of sand flies for more comprehensive investigation. In a previous study, sand flies collected in a leishmaniasis-free area of Thailand also exhibited negative results for *Leishmania* infection, whereas in a leishmaniasis-positive area, a low prevalence (0.45%) of *Leishmania* was detected [19]. In the future, an investigation of larger sand fly populations from several provinces and a variety of locations using massive molecular screening needs to be performed and possible reservoir hosts should be investigated in those areas; such studies will provide additional information on leishmaniasis transmission in Thailand. Finally, future studies on sand fly diversity in Thailand should also consider male specimens, which were not identified in the present study. Indeed, the identification of male specimens could have increased the number of species found, and the exclusion of male specimens from the analysis is a limitation of the study.

## Conclusion

Our study disclosed a high diversity of sand fly species in six provinces of Thailand, highlighting that DNA barcoding is an important method for identifying sand flies. The absence of *Leishmania* spp. DNA in the tested sand flies suggests that a larger number of sand flies should be collected in the future, including in other locations around Thailand, ultimately to monitor the circulation of *Leishmania* spp. and the possible emergence of leishmaniasis in the country.

## Supplementary Information

**Additional file 1: Table S1.** Average temperature, actual rainfall and location coordinate of study sites.

**Additional file 2: Table S2.** Cluster sequence analysis showing the OTUs generated from COI sequences using the Refined Single Linkage (RESL) algorithm.

**Additional file 3: Table S3.** Barcode gap analysis showing the distribution of distances within each species and the distance to the nearest neighbor of each species.

**Additional file 4: Table S4.** Pairwise distance analysis of 68 nucleotide sequences were obtained using the Kimura 2-parameter model in MEGA X.

**Additional file 5: Figure S1.** The amplification curve of *Leishmania* DNA (1 ng) served as a positive control in the reaction. Negative samples were defined by the absence of fluorescent signals above the threshold within 35 cycles.

**Additional file 6: Table S5.** GenBank accession numbers and BIN of this study.

## Data Availability

All sequences were deposited in GenBank and BOLD (Project “OPHU”). GenBank accession numbers and BIN in this study are provided in Additional file 6: Table S5.

## Abbreviations

BIN: Barcode index number
BOLD: Barcode of Life Data System
CL: Cutaneous leishmaniasis
CRI: Chiang Rai
CTI: Chantaburi
K2P: Kimura two-parameter
KRI: Kanchanaburi
NJ: Neighbor-joining
OTU: Operational taxonomic unit
PYO: Phayao
RESL: Refined single linkage algorithm
SKA: Songkhla
SSK: Sisaket
UBN: Ubon Ratchathani
VL: Visceral leishmaniasis
