# Supplementary material for: Sand fly identification and screening for Leishmania spp. in six provinces of Thailand
Source: Parasit Vectors. 2021 Jul 3;14:352. doi: 10.1186/s13071-021-04856-6 (PMC8254935; doi:10.1186/s13071-021-04856-6)
Supplement: Supplementary file 5 — Additional file 5: Figure S1. The amplification curve of Leishmania DNA (1 ng) served as a positive control in the reaction. Negative samples were defined by the absence of fluorescent signals above the threshold within 35 cycles. [file 13071_2021_4856_MOESM5_ESM.pptx]

## Slide 1
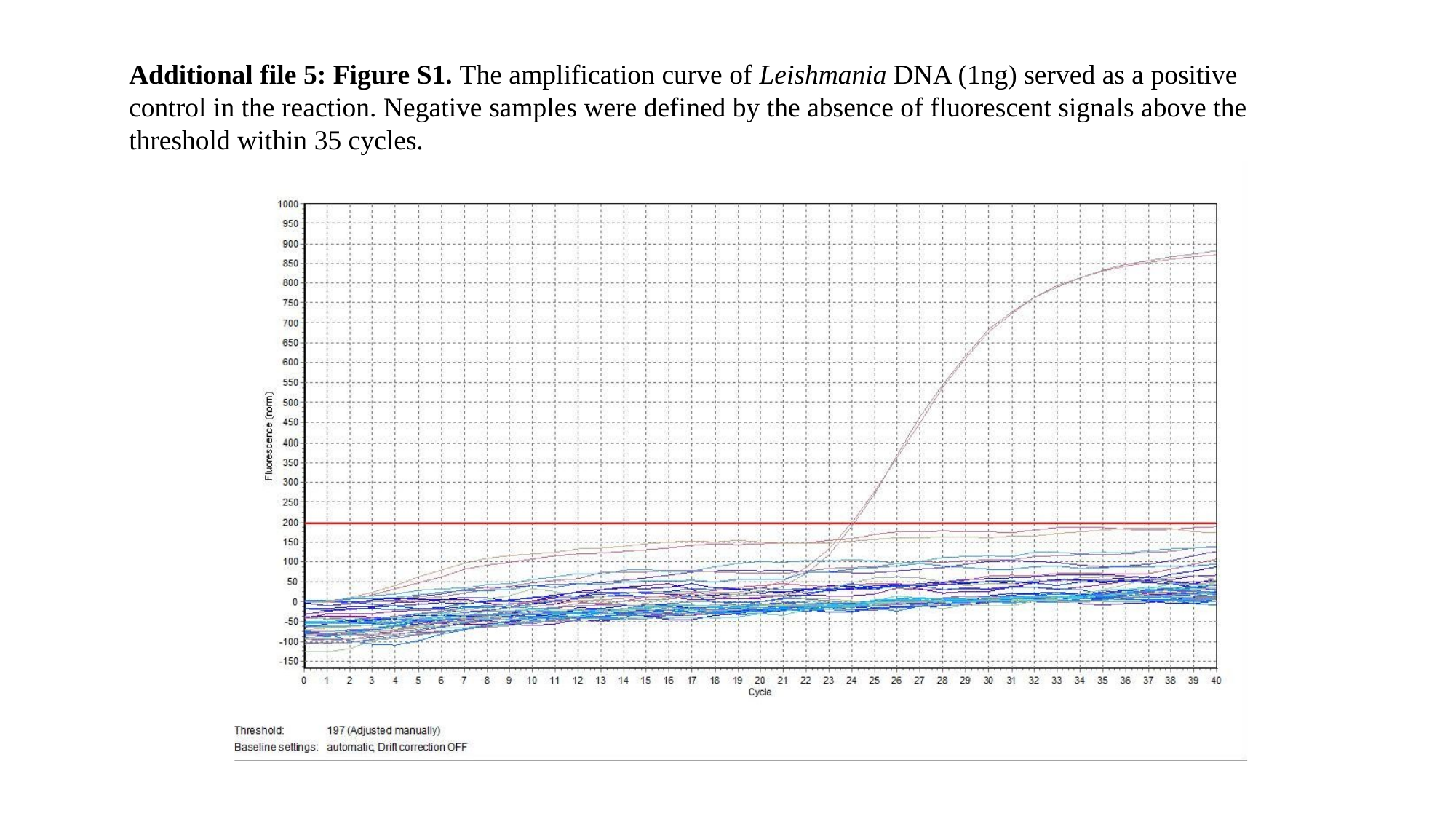

Additional file 5: Figure S1. The amplification curve of Leishmania DNA (1ng) served as a positive control in the reaction. Negative samples were defined by the absence of fluorescent signals above the threshold within 35 cycles.
